# Nationwide Rubella Epidemic — Japan, 2013

**Published:** 2013-06-14

**Authors:** Keiko Tanaka-Taya, Hiroshi Satoh, Satoru Arai, Takuya Yamagishi, Yuichiro Yahata, Kazutoshi Nakashima, Tamie Sugawara, Yasushi Ohkusa, Tamano Matsui, Takehito Saito, Kazuhiko Kanou, Tomoe Shimada, Hitomi Kinoshita, Kazuyo Yamashita, Yoshinori Yasui, Yuki Tada, Yoshio Mori, Makoto Takeda, Tomimasa Sunagawa, Kazunori Oishi, Peter Strebel, W. William Schluter, Hajime Kamiya, Susan E. Reef, Susan Y. Chu, Rebecca Martin

**Affiliations:** National Institute of Infectious Diseases, Japan; Accelerated Control and Elimination of Vaccine Preventable Diseases, World Health Organization, Geneva, Switzerland; Western Pacific Regional Office, World Health Organization, Manila, Phillipines; Div of Bacterial Diseases, National Center for Immunizations and Respiratory Diseases; Global Immunization Div, Center for Global Health, CDC

Rubella usually is a mild, febrile rash illness in children and adults; however, infection early in pregnancy, particularly during the first 16 weeks, can result in miscarriage, stillbirth, or an infant born with birth defects (i.e., congenital rubella syndrome [CRS]) (1). As of 2013, goals to eliminate rubella have been established in two World Health Organization regions (the Region of the Americas by 2010 and the European Region by 2015), and targets for accelerated rubella control and CRS prevention have been established by the Western Pacific Region (WPR) (2). In 1976, Japan introduced single-antigen rubella vaccine in its national immunization program, targeting girls in junior high school. In 1989, a measles-mumps-rubella (MMR) vaccine was introduced, targeting children aged 12–72 months. However, adult males remain susceptible to rubella. From January 1 to May 1, 2013, a total of 5,442 rubella cases were reported through the rubella surveillance system in Japan, with the majority (77%) of cases occurring among adult males. Ten infants with CRS were reported during October 2012–May 1, 2013. Countries and regions establishing a goal of accelerated control or elimination of rubella should review their previous and current immunization policies and strategies to identify and vaccinate susceptible persons and to ensure high population immunity in all cohorts, both male and female.

During 1999–2007, rubella surveillance in Japan consisted of aggregate case reporting to the pediatric sentinel surveillance system. Cases were reported from a representative sample of approximately 3,000 pediatric inpatient and outpatient medical facilities. In January 2008, the sentinel surveillance systems were replaced by nationwide case-based surveillance for rubella, and all physicians were required to report any clinically diagnosed or laboratory-confirmed rubella case* to local health officials. In April 1999, nationwide, case-based surveillance for CRS† had been established.

Until the early 2000s, rubella was endemic in Japan, with periodic epidemics approximately every 5 years and seasonal increases in the spring and summer. The number of reported rubella cases remained at record low levels until 2010, and in 2011, a few outbreaks were reported in the workplace among adult males. In 2012, the number of rubella cases sharply increased to 2,392, with the rise in cases continuing into 2013 (Figure 1). From January 1 to May 1, 2013, a total of 5,442 rubella cases were reported (Table). Of these cases, 3,936 (72.3%) were laboratory confirmed. Geographically, over 60% of rubella cases were reported from Kanto area, in the eastern part of Japan comprised of Tokyo and its surrounding prefectures. In recent weeks, the epidemic has expanded from Kanto to other parts of Japan, including Osaka, Hyogo, Aichi, Fukuoka, and Kagoshima. Of the 5,442 cases, males accounted for 4,213 cases (77.4%), of which 3,878 cases (92.0%) were in persons aged >20 years (Figure 2). Of the 4,834 cases in persons aged >20 years, 1,727 (36%) were in persons aged 30–39 years and 1,535 (32%) in persons aged 20–29 years. Among rubella cases, vaccination history was unknown in a majority of cases (3,538 [65%]). For the 1,904 reported rubella cases with known vaccination status, 1,566 (82%) occurred in persons who had not received rubella vaccine (Table). Virus genotypes were determined for 150 cases in 2012; of these, 123 (82.0%) and 26 (17.0%) were genotypes 2B and 1E, respectively (3).

During 2008–2011, three cases of CRS were reported nationwide. Since October 2012, 10 CRS cases have been reported from Hyogo (two), Aichi (two), Osaka (two), Tokyo (one), Kagawa (one), Saitama (one), and Kanagawa (one). Six of the mothers of infants with CRS had not received rubella vaccine, and four had unknown vaccination history.

Population immunity is measured by administrative coverage and seroprevalence surveys. In 2011, administrative measles-rubella (MR) vaccine coverage was 95.3% at age 1 year, 92.8% at age 5–6 years, 88.1% at age 12–13 years, and 81.4% at age 17–18 years. Population immunity for eight vaccine-preventable diseases is measured by the National Epidemiological Surveillance of Vaccine Preventable Diseases, an annual, national seroepidemiologic survey conducted among a representative sample of the Japanese population. In 2012, 14 prefectures in Japan joined this serologic survey by measuring rubella hemagglutination inhibition antibody levels in 5,094 healthy persons. Among adults aged 30–50 years, seropositivity for rubella antibody (1:8) was 73%–86% among males and 97%–98% among females (4).

In response to the current outbreak, Japan’s Ministry of Health, Labor, and Welfare provided guidance to health-care authorities (5). The guidance is to provide information on rubella disease and CRS for pregnant women and their households and encouraged vaccination of the family members of pregnant women (because rubella vaccine is contraindicated in pregnant women) and vaccination for women who plan to get pregnant. The local governments in approximately 100 cities, including several districts in the Tokyo metropolitan area that had high numbers of reported rubella cases, have provided partial funding to help with the cost of MR vaccine or a single rubella vaccine for women planning pregnancy and for men who are living with a pregnant woman. In addition, mass media agencies in Japan have provided information about the rubella epidemic, including rubella disease and CRS, which has helped increase awareness about the importance of rubella vaccination.

## Editorial Note

The primary purpose of rubella vaccination is to prevent congenital rubella virus infection, including CRS. In WPR, the Immunization Technical Advisory Group endorsed a regional accelerated rubella control and CRS prevention goal to decrease rubella incidence to <10 cases per million population and CRS incidence to <10 cases per million live births each year by 2015 (6). In 2012, Japan reported 18.7 rubella cases per million population, a rate higher than the WPR annual incidence target. As of May 2013 (4 months into the year), the number of reported rubella cases is already double the total number of cases in 2012.

In 1976, Japan established a goal to prevent CRS and introduced single-antigen rubella vaccine in its national immunization program, targeting girls in junior high school. In 1989, an MMR vaccine was introduced, targeting children aged 12–72 months, but this combination vaccine was withdrawn in 1993 after reports of aseptic meningitis related to the mumps component. In 1995, vaccination policy was changed to make all vaccines strongly recommended but not mandatory, and in 2006, the MR combined vaccine was introduced, with a 2-dose schedule administered at 1–2 years and 5–7 years. After a large measles outbreak in 2007 and 2008, a catch-up MR vaccination program was implemented, targeting two age cohorts (those aged 12 years and those aged 17 years) each year during 2008–2013 to ensure high population immunity among persons aged 12–22 years in 2013.

What is already known about this topic?

Congenital rubella syndrome (CRS) is caused by fetal infection with rubella virus from the mother and is characterized by birth defects such as hearing impairment, heart defects, and cataracts. Several countries that initially vaccinated only adolescent or adult women, then later introduced rubella vaccine into their routine programs or conducted mass campaigns in adolescent and adult females, have experienced large rubella outbreaks among adolescent and young adult males, with a concomitant increase in infants with CRS.

What is added by this report?

In 2012, the number of rubella cases in Japan sharply increased to 2,392, with the rise in cases continuing into 2013 and resulting in a cumulative total of 5,442 cases from January 1 to May 1, 2013. Of these cases, 72% were laboratory confirmed, and 23% were in females. Since October 2012, 10 CRS cases have been reported.

What are the implications for public health practice?

Countries using rubella vaccine should aim to prevent rubella outbreaks (i.e., achieve and maintain interruption of rubella virus transmission) by ensuring high rubella immunity across all age groups (both males and females). In cohorts born since the introduction of rubella vaccine, this immunity is achieved primarily through uniformly high vaccination coverage.

In the current outbreak, males aged 20–39 years, who were not included in the initial rubella vaccination program, accounted for 68% of the reported cases. However, with the introduction of 2 doses of MR vaccine into the national vaccination schedule in 2006 for both boys and girls and the successful catch-up vaccination program, children who currently are aged <15 years account for only 5.6% of the cases. In other countries (e.g., Brazil, Chile, and Argentina), where only adolescent or adult females have been targeted through national immunization programs or as part of mass vaccination campaigns, similar large outbreaks have occurred among adolescent and adult males, with a concomitant increase in CRS cases. These types of outbreaks emphasize that national immunization programs should ensure high levels of immunity in all cohorts born since the introduction of rubella vaccine (both males and females) either through the routine program or high-quality mass campaigns that are sufficient to interrupt rubella virus transmission and prevent CRS cases. In addition, programs should implement high-quality, case-based rubella and CRS surveillance and respond promptly and rapidly to outbreaks.

The effects of this outbreak have been wide-ranging, both within Japan and internationally. In the Region of the Americas, where endemic rubella virus transmission has been interrupted, importations have occurred in the United States and Canada in 2013. The international spread of rubella virus from Japan provides a reminder that countries in regions that have eliminated rubella need to maintain high levels of vaccination coverage and high-quality surveillance to limit the spread and detect imported rubella virus.

## Figures and Tables

**FIGURE 1:**
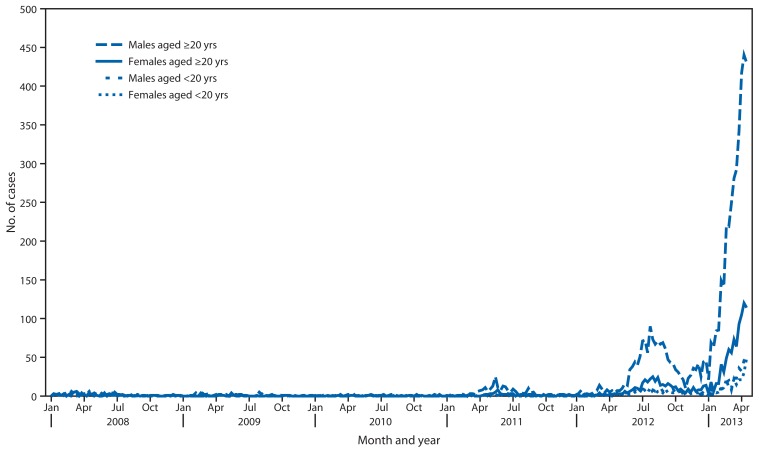
Number of rubella cases, by sex and age group — Japan, 2009–2013^*^ ^*^ As of April 24, 2013.

**FIGURE 2:**
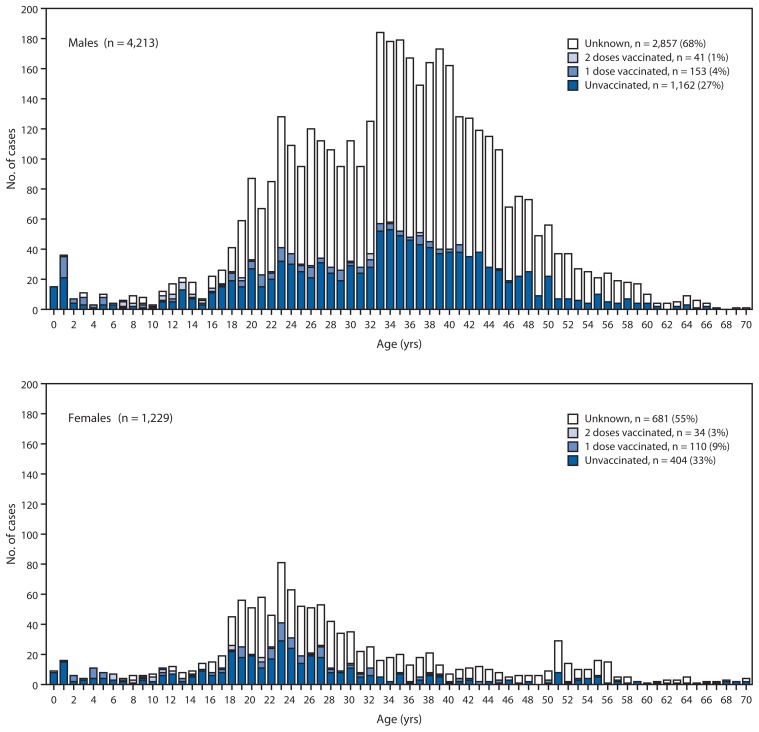
Number of rubella cases among males and females, by age and vaccination history — Japan, surveillance week 1 to 17, 2013^*^ ^*^ As of May 1, 2013.

**TABLE:** Number and percentage of rubella cases, by year and selected characteristics — Japan, 2009–2013

Characteristic	2009		2010		2011		2012		2013*	
				
	No.	(%)	No.	(%)	No.	(%)	No.	(%)	No.	(%)
**Total**	**147**	**(100)**	**87**	**(100)**	**378**	**(100)**	**2,392**	**(100)**	**5,442**	**(100)**
Rubella cases per 1,000,000 population	1.2		0.7		3.0		18.7		42.5	
**Sex**										
Male	98	(66.7)	54	(62.1)	278	(73.5)	1,797	(75.1)	4,213	(77.4)
Female	49	(33.3)	33	(37.9)	100	(26.5)	595	(24.9)	1,229	(22.6)
**Age group (yrs)**										
<1	4	(2.7)	1	(1.1)	2	(0.5)	16	(0.7)	24	(0.4)
1–4	22	(15.0)	11	(12.6)	23	(6.1)	69	(2.9)	94	(1.7)
5–9	13	(8.8)	10	(11.5)	10	(2.6)	37	(1.5)	68	(1.2)
10–14	17	(11.6)	8	(9.2)	18	(4.8)	56	(2.3)	118	(2.2)
15–19	19	(12.9)	5	(5.7)	29	(7.7)	217	(9.1)	304	(5.6)
20–29	22	(15.0)	20	(23.0)	114	(30.2)	741	(31.0)	1,535	(28.2)
30–39	30	(20.4)	16	(18.4)	94	(24.9)	681	(28.5)	1,727	(31.7)
40–49	13	(8.8)	14	(16.1)	59	(15.6)	430	(18.0)	1,103	(20.3)
50–59	4	(2.7)	1	(1.1)	22	(5.8)	124	(5.2)	396	(7.3)
>59	3	(2.0)	1	(1.1)	7	(1.9)	21	(0.9)	73	(1.3)
**Diagnosis**										
Clinically diagnosed	63	(42.9)	26	(29.9)	83	(22.0)	599	(25.0)	1,506	(27.7)
Laboratory confirmed	84	(57.1)	61	(70.1)	295	(78.0)	1,793	(75.0)	3,936	(72.3)
**Vaccination status**										
Unvaccinated	46	(31.3)	17	(19.5)	96	(25.4)	605	(25.3)	1,566	(28.8)
Once	41	(27.9)	14	(16.1)	29	(7.7)	180	(7.5)	263	(4.8)
Twice	4	(2.7)	4	(4.6)	9	(2.4)	49	(2.0)	75	(1.4)
Uncertain	56	(38.1)	52	(59.8)	244	(64.6)	1,558	(65.1)	3,538	(65.0)
Total CRS* **cases**	**2**	**(100)**	**0**	**—**	**1**	**(100)**	**5**	**(100)**	**5**	**(100)**
CRS cases per 1,000,000 live births	2.0		0.0		1.0		4.8		4.8	

**Abbreviation:** CRS = congenital rubella syndrome.

* As of May 1, 2013.

## Footnotes

* Rubella case definition: clinically diagnosed rubella case is a diffuse punctate and maculopapular rash, fever, and lymphadenopathy; laboratory-confirmed rubella case is the presence of all of the mentioned signs and one of the following: 1) isolation of the virus or detection of viral RNA from blood, throat, or cerebrospinal fluid samples by reverse transcription–polymerase chain reaction; or 2) detection of rubella-specific immunoglobulin M antibodies from a serum sample or a significant increase in rubella-specific immunoglobulin G antibody titers in paired serum samples obtained at acute and convalescent phases.

† Laboratory-confirmed CRS case definition: 1) clinically confirmed CRS in an infant who has a positive blood test for rubella-specific immunoglobulin M or hemagglutination inhibition antibody levels sustained or higher than expected from passively transferred maternal antibody; or 2) detection of rubella virus in specimens from throat, saliva, or urine. CRS is clinically confirmed if an infant has 1) at least two of the following complications: cataract, congenital glaucoma, congenital heart disease, hearing impairment, or pigmentary retinopathy; or 2) one of those complications and one of the following complications: purpura, splenomegaly, microcephaly, meningoencephalitis, radiolucent bone disease, or jaundice developed within 24 hours after birth.
